# Early Communication Behaviors in Infants With Cleft Palate With and Without Robin Sequence: A Preliminary Study

**DOI:** 10.1177/10556656211031877

**Published:** 2021-07-14

**Authors:** Stephanie van Eeden, Yvonne Wren, Cristina McKean, Helen Stringer

**Affiliations:** 1 School of Education, Communication and Language Sciences, 5994 Newcastle University, Newcastle upon Tyne, United Kingdom; 2 Newcastle upon Tyne Hospitals NHS Foundation Trust, Newcastle upon Tyne, United Kingdom; 3 Bristol Speech and Language Therapy Research Unit, North Bristol NHS Trust, Bristol, United Kingdom; 4 1980 University of Bristol, Bristol, United Kingdom

**Keywords:** Robin sequence, cleft palate, communication, language, speech, infant, Cleft Collective

## Abstract

**Objective:**

To investigate the early communication behaviors in infants with nonsyndromic isolated cleft palate (iCP) and Robin sequence (RS).

**Design:**

Group comparison using parent report.

**Participants:**

There were 106 participants included in this study. Two groups were selected from the UK Cleft Collective resource. Parents had completed the Language ENvironment Analysis Developmental Snapshot questionnaire when their child turned 13 months. There were 78 participants in the iCP group and 28 in the RS group.

**Main Outcome Measure(s):**

Total number of communication behaviors reported on the questionnaire. Subdomains for expressive and receptive language and social communication behaviors were also analyzed.

**Results:**

There were no statistically significant group differences. Parents of infants with RS reported fewer later communication behaviors compared to the iCP group. Infants in both groups had fewer communication behaviors compared to the normative sample. Across the whole sample, post hoc analysis revealed a significant correlation between severity of the cleft and social communication behaviors and expressive but not receptive language. Infants with a cleft of the hard and soft palate were more likely to be in the RS group (odds ratio: 7.04 [95% CI: 1.55-32.04]; *P* = .01).

**Conclusions:**

Both groups reported similar levels of early communication. Some divergence of more complex language skills was seen, although there were no significant group differences. A relationship with the diagnosis of a cleft of the hard or soft palate with expressive language behaviors was found. Further study into the impact of cleft severity on early speech development and the relationship with later language skills is needed along with longitudinal follow-up of this population.

## Introduction

Robin sequence (RS) or Pierre Robin sequence is a low incidence condition with high clinical need (Paes et al., 2015). The presentation of RS is well-documented in the literature (Robin, 1923; Figueroa et al., 1991; Gangopadhyay et al., 2012), but there is often disagreement about the definitive diagnosis. An international consensus meeting held in the Netherlands in 2014 agreed upon the following definition: micrognathia, glossoptosis, and airway obstruction, with or without cleft palate (Breugem et al., 2016). This article focuses on a group of infants with nonsyndromic RS and cleft palate and compares outcomes to infants with cleft palate alone or isolated cleft palate (iCP).

An outcome of key importance to families and clinicians alike is the speech development of the child with cleft palate with or without RS. Consequently, speech difficulties in this population are extensively described in the literature (Howard & Lohmander, 2011; Vallino et al., 2019). The most commonly occurring relate to resonance and nasal airflow errors (Kummer, 2011) and deviant articulatory patterns which develop to compensate for a faulty velopharyngeal mechanism (Britton et al., 2014). It is increasingly understood that children born with RS in addition to having a cleft palate may have poorer speech outcomes than those with iCP (Stransky et al., 2013; Hardwicke et al., 2016). Most recently, the national audit registry report on outcomes in cleft lip and palate in the United Kingdom showed that 31.7% of children aged 5 years with RS still had significant articulation difficulties, compared with only 17.7% of those with iCP (CRANE, 2020). Children with RS often have wide clefts of the hard and soft palate and can have their surgical palate repair later than those children with iCP (Godbout et al., 2014; Logjes et al., 2021). Both issues have been reported to lead to poorer speech outcomes (Lam et al., 2012; Pasick et al., 2014).

Around 30% of children with RS also have an additional syndromic diagnosis, with Stickler syndrome, 22q11 deletion syndrome, and fetal alcohol syndrome being most frequently reported (Izumi et al., 2012; Levaillant et al., 2017; Karempelis et al., 2020). Additional syndromic diagnoses will impact on speech and language outcomes for these children. However, cases with nonsyndromic RS are also reported to have additional cognitive and psychosocial difficulties (Kapp-Simon & Krueckeberg, 2000; Drescher et al., 2008; Filip et al., 2015; Alencar et al., 2017). The reasons for this are unclear. Hypotheses have been postulated regarding the effect of oxygen desaturation due to prolonged airway difficulties (Almajed et al., 2017), disturbed sleep in infancy (Ehsan et al., 2019), neurological deficits (Abadie et al., 2002), and repeated anesthesia in infancy (O’Leary & Warner, 2017). Early language and communication development are key components of children’s cognitive and psychosocial development and the relationship between these and speech development have been rarely investigated.

### Speech and Language Difficulties in RS With Cleft Palate

In a systematic review of the literature between 1966 and 2014, Wan et al. (2015) found 6 articles which considered speech outcomes in RS in comparison to iCP (Lehman et al., 1995; Witt et al., 1997; Khosla et al., 2008; Goudy et al., 2011; Stransky et al., 2013; Black & Gampper, 2014). These articles reported conflicting results. For example, Stransky et al. (2013) reported a significant difference in velopharyngeal function between children aged 8 years with RS and those with iCP; where the other 4 articles which measured this outcome found no significant group differences. Only 3 articles studied articulation outcomes (Lehman et al., 1995; Khosla et al., 2008; Stransky et al., 2013). All found no significant group differences. However, there were limited descriptions of the types of error patterns. The review’s authors conclude that 5 of the 6 articles were methodologically flawed due to small sample sizes (reporting between 11 and 55 cases of RS), poor follow-up, and not separating syndromic from nonsyndromic cases. Since Wan et al.’s review, participant numbers have improved in some studies (reporting between 24 and 96 cases of RS) and results suggest more group differences between children with RS and those with iCP. Filip et al. (2015) in their retrospective assessment of 93 children with nonsyndromic RS with cleft palate found that 33.3% needed secondary surgery for velopharyngeal incompetence (VPI), compared to 19.4% in the iCP control group; 46.7% had cleft-related articulation difficulties (no comparison with the control group is reported). However, no specific age at outcome was reported, making it difficult to compare with other studies. Similar rates of VPI have been found in other studies. For example, Morice et al. (2018) found a 30.5% VPI rate in their isolated RS group. Hardwicke et al. (2016) reported rates as high as 46% for hypernasality. Their matched case study showed significant group differences between children aged 5 years with RS and those with iCP on all outcome measures, with increased likelihood of need for secondary surgery (odds ratio [OR]: 7.85 [95% CI: 1.5-41.3]).

Despite increased interest in the comparison of speech outcomes in children with RS compared to those with iCP, very few studies have investigated the language skills of children with RS. There are 2 published studies; neither report comparisons to an iCP group. Smith et al. (2014) studied outcomes at 3 years of age in relation to sleep disturbance caused by obstructive sleep apnea in children with cleft palate and RS. They found significantly lower scores in both receptive and expressive language using the Bayley Scales of Infant and Toddler Development-III (Bayley, 2006) when compared to children with iCP. Thouvenin et al. (2013) reported one of very few longitudinal studies in any research into speech and language in this population. Developmental assessment was carried out at 15 months, 3 years, and 6 years using the Brunet-Lezine test (a French test similar to the Bayley Scales of Infant Development [Josse, 1997]) and the Kaufman Assessment Battery for Children (Kaufman & Kaufman, 1993) in the older age-groups. They studied 27 children with nonsyndromic RS and 12 with RS and Stickler syndrome. They found delays in the early years, reporting poor language skills in 26% of 15-month-olds. However, the mean language score on the scale for the infants with nonsyndromic RS was 93.4 (range 82.4-104.4), indicating language scores within the normal range as a group. At 3 years of age, they report 46% of children falling below 1 standard deviation (SD) on vocabulary measures, with a mean standard score of 99.7. This suggests that there were some children with very low scores and others with very high scores, but the range is not reported. Unfortunately, follow-up at 6 years was only for global developmental scores, which had improved over time; no comparison of language scores was reported.

Research examining speech and language in this population to date has been inconsistent in its methodology. There has been a lack of consensus about diagnostic criteria leading to heterogeneous groups. Participant numbers have been small, and the lack of longitudinal data makes it difficult to understand the progression of speech, language, and communication skills in children with RS. In contrast, studies of isolated cleft lip and palate have investigated early babble, vocabulary, and language development to a much greater extent (Lohmander-Agerskov et al., 1994; Scherer & D’Antonio, 1995; Jocelyn et al., 1996; Chapman et al., 2001; Willadsen & Albrechtsen, 2006; Hardin-Jones & Chapman, 2014). Other studies have shown ongoing language problems (Morris & Ozanne, 2003) and linked this to other linguistic skills such as reading (Chapman, 2011) and speech (Pamplona et al., 2000). For speech and language pathologists to begin to understand how best to treat children with RS and cleft palate, it is necessary to gain a greater understanding of their developmental trajectory with regard to language, communication, and speech and how this might differ from children with iCP. This will in turn influence caseload management and allocation of resources.

### Early Communication Measures From Cohort Studies

Gathering data on early language skills in infants through direct assessment or observation on a large-scale can be time-consuming and difficult (Dale et al., 1989). Therefore, parental report is commonly used for assessment of language in preschool children. This method has been used in many cohort studies across the world (Magnus et al., 2006; Fraser et al., 2013; Reilly et al., 2018). There are differing views regarding the validity and reliability of parent report. Many studies of toddlers aged 2 to 3 years have found moderate to high correlations between parent report and direct assessments with most reporting correlation coefficients in the range of *r* = .48 to .87 for expressive language (Dale et al., 1989; Rescorla & Alley, 2001; Feldman et al., 2005; Sachse & Von Suchodoletz, 2008). A true picture of receptive language skills is frequently reported as more challenging to capture, with the range of correlations reported in these same studies much broader (*r* = .13-.75). From the cleft lip and palate literature, Scherer & d’Antonio (1995) studied 60 children (30 with cleft palate and 30 without) with a mean age of 24 months and compared the results of parent report with a range of direct language assessments. Strong correlations were seen between parent reports of vocabulary/length of sentences and mean length utterance measured by the speech and language pathologist (*r* = .81, *P* < .01) and moderate correlations from the questionnaire and expressive language/vocabulary (*r* = .57-.62, *P* < .01).

The use of parental report and large prospective data registries collected over time offer a pragmatic and viable method of collecting valid and reliable data at sufficient scale for meaningful analyses to be completed. This study used data gathered by a large national cohort study in the United Kingdom, the Cleft Collective, to identify a relatively large and representative sample to explore early communication development in infants with nonsyndromic cleft palate with and without RS and to examine the differences between these 2 groups.

## Ethics

Ethical approval for this study was granted by the University Ethics Committee at the lead author’s host university.

## Aims

This study aims to explore the early presentation of communication abilities in infants with nonsyndromic RS with cleft palate and to compare that presentation to children with iCP. Studies of older children have reported deficits in social communication (Filip et al., 2015), expressive language (Thouvenin et al, 2013), and receptive language (Smith et al., 2014). To that end, we address the following questions.

- Do infants with nonsyndromic RS exhibit fewer communication behaviors than peers with iCP?
- Are there differences across the subdomains of expressive or receptive language skills and social communication?
- Are the patterns seen suggestive of clinical levels of difficulty?

## Methods

### Design

This is an exploratory study of initial data taken from the Cleft Collective cohort study (see below). It is a group comparison study. All data from participants that met the inclusion criteria were analyzed. A matched case study was considered initially using one-way analysis of variance (ANOVA). This reduced the number of participants in the study to 44 and as the results of this initial analysis did not differ from those using the methods in this final study, the authors decided to include and present all data available for this exploratory study.

### Data Collection

#### The Cleft Collective

The Cleft Collective cohort study is a large study in the United Kingdom collecting data from all children born with cleft lip and/or palate. Since 2013, data on over 9000 participants have been collected. This includes a range of data that are of interest to researchers in the area of cleft, including genetic samples, details of operations, and data on socioeconomic status and family circumstances and parental opinion on outcomes such as speech, language, and education. Further details are available on the website http://www.bristol.ac.uk/cleft-collective. The Cleft Collective Speech and Language (CC-SL) study is a nested study within the larger Cleft Collective cohort study. Parents of children born with a cleft affecting the palate are asked to consent to this study if they are part of the main cohort study. Data on speech and language outcomes are currently gathered at 13 months, 18 to 24 months, and 3 years.

#### Participants

The sample was taken from children participating in the CC-SL study. At the point of data analysis, 393 questionnaires had been sent out to parents of children born with a cleft affecting the palate. This included children with a diagnosis of cleft lip and palate. The return rate was 85.7% (n = 294). The following inclusion criteria were used: all participants with iCP with or without RS who had complete parent report data from the questionnaire sent at 13 months. All those with unilateral or bilateral cleft lip and palate (n = 153), an additional syndromic diagnosis (n = 15), or incomplete questionnaire data (n = 20) were excluded. There were 106 participants in total: 78 with iCP (iCP group) and 28 with cleft palate and RS (RS group). There was a range of ages at time of data collection from 13 to 19 months. Consideration was given to excluding participants over the age of 16 months (6 from the iCP group and 1 from the RS group). However, as there were no significant differences across the groups in terms of age at time of data collection (*P* = .20), and analysis of results was not affected, data from all participants are reported. The mean age of participants was 14 months.

#### Procedures

The study used maternal responses to the Language ENvironment Analysis Developmental Snapshot (LDS) questionnaire (Gilkerson et al., 2017). This questionnaire was designed by speech-language pathologists, linguists, and statisticians at the Language ENvironment Analysis (LENA) institute. It asks 52 age-ordered questions relating to communication and language development and was designed to be used with parents of children aged 2 to 36 months. Parents are asked to rate *yes* or *no* to indicate whether their child has achieved the behavior stated. There are normative data from a sample of children living in the United States, and the tool has also been validated against other questionnaires used in this field (eg, the MacArthur-Bates Communicative Development Inventories [Fenson et al., 2007]). Construct validity has been confirmed through correlating outcomes from the LDS and direct language assessments such as the Preschool Language Scales-4 and the Receptive-Expressive Emergent Language Test-3. Correlations were high (*r* = .81-.96, average *r* = .93). The LDS was sent out to parents in the CC-SL study to complete when their babies turned 13 months of age along with a device to record babble patterns. These were returned in a prepaid envelope to the Cleft Collective study center. Reminders to return the questionnaires were sent out by the Cleft Collective as a standard practice to help increase the rate of return.

#### Outcome measures

The primary outcome measure was the total number of communication behaviors out of 52 recorded by parents. In order to further explore the patterns of communication behaviors, the lead author grouped the questions into subdomains. These included social communication behaviors which indicated early vocalization and babble behaviors in a social context and expressive and receptive language behaviors (see Supplemental Appendix). Data on potential confounding variables held by the Cleft Collective from the main cohort study were also added to the data set. These included measures of mother’s level of education divided into 3 levels (1 = standard level or below [General Certificate of Secondary Education taken at 16 years in the United Kingdom]; 2 = advanced level [A-levels taken at 18 years]; 3 = first degree level or above). This was used as a proxy for socioeconomic status which has been found to be associated with language outcomes in several studies (Reilly et al., 2007; Miser & Hupp, 2012; Law et al., 2013). Although there is less evidence of association with language outcomes, history of diagnosed hearing loss and gender of the child were also chosen as possible confounders (Schlieper et al., 1985; Feldman et al., 2000; Norbury et al., 2016). A further variable measuring the severity of the cleft palate was included (soft palate only vs hard and soft palate cleft). This has been found to impact speech development in other studies but has not been investigated in terms of communication outcomes (Lam et al., 2012). Age at cleft palate repair was also included; later palate repair has been reported to delay babble patterns and early expressive language (Chapman et al., 2008).

### Data Analysis

IBM SPSS Version 26 software was used for all statistical analysis. Descriptive statistics for the sample, primary outcome measure, and subdomains are reported. To investigate the potential clinical levels of difficulty, comparisons from both groups with reported norms from the LDS are also reported as well as the percentage of participants in each group falling below 1.2 SDs from the mean for this cohort. This is in line with Records & Tomblin. (1994) who showed this was the cutoff level at which speech and language pathologists diagnosed language impairment. Means and SDs for the primary outcome measure and the 3 subdomains are reported. Data were normally distributed. Group differences for outcome measures were analyzed using a one-way ANOVA and a 2-tailed Fisher exact test to analyze group differences for individual behaviors. Linear regression analysis was carried out to investigate the influence of a diagnosis of RS, history of hearing loss, gender, socioeconomic status, age at palate repair, and severity of the cleft on the primary outcome measure. A level of significance of *P* <.05 was used in all tests. Missing data were accounted for on a pairwise or analysis-by-analysis basis in SPSS. This enabled as much data as possible to be included, excluding only those where the missing data were pertinent to the calculation (Field, 2009).

## Results

### Sample

There were no group differences in the sample in terms of history of hearing loss (*P* = .81), mother’s level of education (*P* = .83), or gender (*P* = .34). The groups did differ in terms of severity of the cleft (*P* < .01) and age at which the palate was repaired (*P* = .02). Of those children with RS, 93% had a cleft of the hard and soft palate, compared to 65% of the iCP group. A binary logistic regression showed that the severity of the cleft significantly predicted which group the child would belong to (OR: 7.04 [95% CI: 1.55-32.04], *P* = .01). There were large amounts of missing data for the following confounding variables: mother’s level of education, age at time of palate repair, and history of diagnosed hearing loss (see Table 1). A chi-square calculation showed missing data percentages only differed across the 2 groups for age at time of palate repair (χ^2^_1_ [n = 105] = 5.35, *P* = .02).

**Table 1.** Descriptive Statistics of the Sample Population.

		n	Mean	Median	Std. deviation	Minimum	Maximum	%
ICP group	Age at time of data collection (months)	78	14.3	14.1	1.1	13.0	19.5	–
	Age at time of palate repair (months)	56	9.4	9.0	2.7	6.0	24.0	–
	Mother’s level of education (levels 1-3)	54	2.52	3	0.75	1	3	–
	Male gender	78	–	–	–	–	–	46
	History of hearing loss	51	–	–	–	–	–	49
	Cleft of the hard and soft palate	74	–	–	–	–	–	65
RS group	Age at time of data collection	28	14.0	13.7	0.8	12.7	16.2	–
	Age at time of palate repair	26	10.9	11.0	2.3	7.0	17.0	–
	Mother’s level of education (levels 1-3)	21	2.48	3	0.81	1	3	–
	Male gender	28	–	–	–	–	–	36
	History of hearing loss	23	–	–	–	–	–	52
	Cleft of the hard and soft palate	28	–	–	–	–	–	93

Abbreviations: iCP, isolate cleft palate; RS, Robin sequence; Std deviation, standard deviation.

### Analysis

#### Total communication behaviors

The RS group had a lower mean for the primary outcome measure of total communication behaviors (16.75 compared to 17.74 for the iCP group); this was not a statistically significant difference (*P* = .35). The normative sample for the LDS (Gilkerson et al., 2017) reported a mean of 22 total communication behaviors at 14 months. Both the iCP and the RS groups were lower than this in this study. The normative sample had a range of 16 to 32 across the ages of 13 to 19 months, which reflects our sample here. This compares to 8 to 37 in the iCP group and 9 to 26 in the RS group. Parents from both the groups in this study reported a larger range of rates of communication behaviors, with much lower minimum scores compared to the normative group.

#### Subdomains

**Figure 1.**
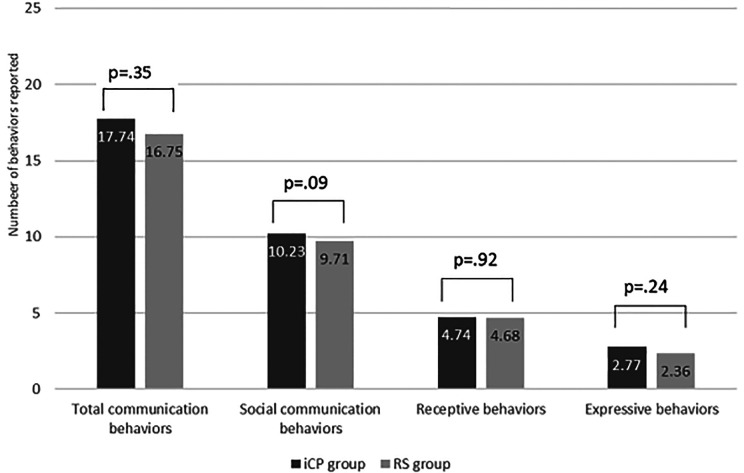
Mean average scores for communication behaviors reported from the LDS questionnaire in both groups. LDS indicates Language ENvironment Analysis Developmental Snapshot.

For social communication behaviors, the RS group had a mean of 9.71 compared with 10.23 in the iCP group (*P* = .09). For receptive language behaviors, the RS group had a mean of 4.68 compared to 4.74 in the iCP group (*P* = .91). For expressive behaviors, the RS group had a mean of 2.36 compared with 2.77 in the iCP group (*P* = .24). All descriptive statistics are reported in Figure 1.

#### Individual questions

**Figure 2.**
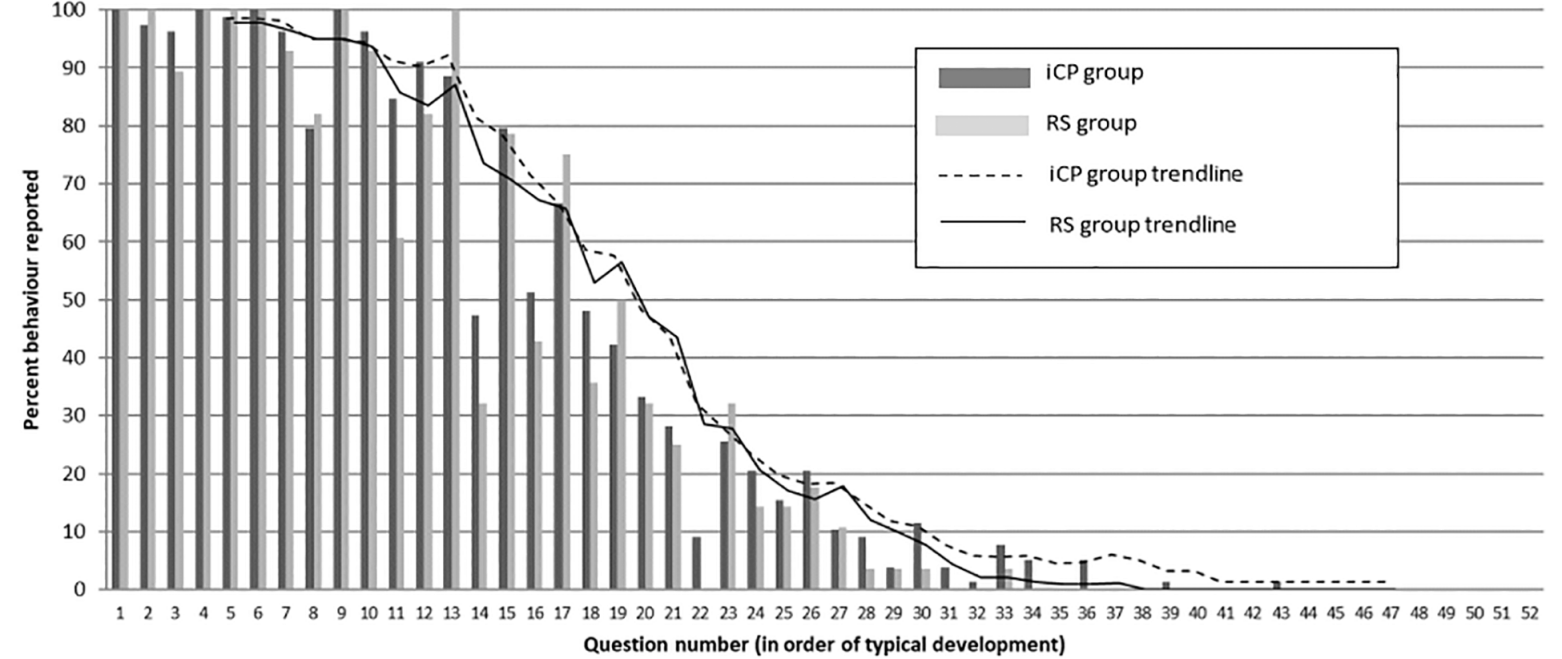
Responses to individual questions by group showing trends.

Analysis of responses to individual questions showed only one to differ between the groups. Parents in the RS group reported a significantly lower level of imitating sounds (question 11—iCP, n = 66 [85%]; RS, n = 17 [61%]; *P* = .02). The results of this study showed no group differences for earlier comprehension tasks such as understanding body parts (iCP, n = 22 [28%]; RS, n = 7 [25%]; *P* = .80) or pointing to specific objects when asked (iCP, n = 52 [67%]; RS, n = 21 [75%]; *P* = .65). In some cases, the RS group performed better. However, for the 4 more complex language tasks—understanding longer instructions, understanding concepts such as color, expressive vocabulary of 10 words, and combining words—none of the RS group were reported to have reached this level. This compared to a few of the iCP group (n = 4-7), but there were no significant group differences for any of the behaviors (*P* = .18-.56). Closer inspection of individual data showed that these higher level behaviors were not always reported by the parents of infants in the older age bracket (>16 months). Indeed, 6 of the 7 who were older than 16 months were not reported to have exhibited any of these behaviors, and the 1 infant who was 19 months at time of data collection was reported only to be exhibiting one of these 4 behaviors (expressive vocabulary of 10 words). This is represented in Figure 2 which shows divergence as more complex behaviors are reported.

#### Clinical levels of difficulty

The SD from this cohort was 4.81. Reports of fewer than 12 communication behaviors represented 1.2 SD below the mean. The number of infants falling below this was 4 in both groups; this represented 14% of the RS group and 5% of the iCP group. This was not a statistically significant difference (χ^2^_1_ [n = 106] = 2.47; *P* = .11).

#### Inferential statistics

Further analysis looked at the influence of all variables together as predictors of outcome (see Table 2). No variable was found to have a significant effect on the primary outcome measure. A diagnosis of RS could not predict the number of total communication behaviors that was reported by parents. Confounding variables of history of hearing loss, gender, mother’s level of education, age at primary repair, and whether a child had a cleft of the soft or hard and soft palate also had no significant effect on the primary outcome measure. Missing data were accounted for pairwise in this analysis. Further analysis using imputed data did not alter the results.

**Table 2.** Linear Regression Analysis for Effects of Variables on the Number of Total Communication Behaviors Reported.^a^

	Unstandardized coefficients	SE	Standardized coefficients	*t*	Significance *P*
(Constant)	22.098	4.192		5.271	.000
Child has RS	−0.355	1.585	−0.033	−0.224	.824
History of diagnosed hearing loss	−1.071	1.382	−0.112	−0.775	.442
Gender	1.962	1.373	0.203	1.429	.159
Mother’s highest educational qualification	−0.738	0.947	−0.116	−0.779	.440
Cleft type (soft vs hard palate)	−1.621	1.545	−0.151	−1.049	.299

Abbreviations: RS, Robin sequence; SE, standard error.

^a^ *r* ^2^ = .123.

#### Post hoc analysis

In the process of exploring the linear regression analysis, some interesting significant correlations stood out. Most notably, there was a significant negative correlation between the severity of the cleft and the total number of communication behaviors reported. Parents of infants who had a cleft of the hard and soft palate reported fewer communication behaviors than parents of those with a cleft of the soft palate only (*r* = .22, *P* = .02). Further analysis of this predictor’s contribution to communication outcomes was carried out post hoc. This showed significant correlations with the severity of the cleft and expressive (*r* = −.22; *P* = .02) and social communication behaviors (*r* = −.27; *P* < .01), but not receptive language behaviors (*r* = .11, *P* = .27).

## Discussion

This article reports the results from a preliminary descriptive study investigating early communication behaviors in infants with cleft palate with and without associated RS. It used data from a parent-reported questionnaire gathered when infants were on average 14 months old. The study aimed to investigate whether infants with nonsyndromic RS exhibited fewer communication behaviors than peers with iCP, and whether there were any early indicators of difficulties with social communication, expressive language, or receptive language.

No group differences were seen in any early communication behaviors. The mean scores for expressive, receptive, and social communication skills were similar whether an infant had a cleft palate with or without nonsyndromic RS. However, parents of infants in the RS group were less likely to report more complex language behaviors, such as understanding longer sentences or concepts. Expressive language skills showed the greatest variation between the groups with the maximum number of behaviors reported to be 6 in the RS group and 11 in the iCP group. Again, more advanced skills such as an expressive vocabulary of more than 10 words or the ability to combine words were not reported at all in the RS group. This study showed 14% of the RS group to have a clinically low level of communication, using 1.2 SDs as the cutoff. This compared to 5% of the iCP group. However, the small numbers, particularly in the RS group, make it difficult to draw any conclusions. Furthermore, the iCP group had 6 infants older than 16 months compared to only 1 in the RS group which may account for this finding, although analysis of the questions representing more complex language skills showed the older children were not always the ones to have achieved these later skills. One other study to date has investigated communication skills in infants with RS. Although they had no comparison group, Thouvenin et al. (2013) showed on average that communication skills at 15 months fell within the normal range, but with 26% falling below 1 SD from the norm. The results from this study would support these findings. They also had small numbers (n = 39) and included 12 participants with Stickler syndrome.

Comparisons of the data on early communication skills from this study with normative data show that both groups reported fewer communication skills. The range of behaviors reported by parents in the LDS normative group across the same age range as in this study (13-19 months) showed the lowest number reported was 16 (Gilkerson et al., 2017). This compares with 8 (iCP group) and 9 (RS group) in this study. Normative data on expressive vocabulary in the United Kingdom show that children on the 50th centile at 14 months have an expressive vocabulary of 12 words (Alcock et al., 2020). In this study, none of the RS group had reached a vocabulary of more than 10 words and only 9% (n = 7) of the iCP group. It would appear then that while there was no evidence of any differences in early communication behavior between infants with nonsyndromic RS and those with iCP, all infants with a diagnosis of nonsyndromic cleft palate may be at risk of slower communication development than their peers. Early language delay in children with cleft palate is well-documented in the literature (Scherer & D’Antonio, 1995; Jocelyn et al., 1996; Hardin-Jones & Chapman, 2014). The underlying reasons for this are unclear. One argument is the relationship between the lack of babble practice in the months prior to cleft palate repair and its impact on speech sound development and subsequent early vocabulary (Scherer et al., 2008). In their matched case study of children with and without cleft palate, Scherer et al. found that babies who had a higher level of babbling at 6 months presented with greater consonant inventories and expressive vocabularies at 30 months.

Evidence to date comparing outcomes in children with RS to those with iCP is strongest with regard to velopharyngeal function and speech outcomes (Filip et al., 2015; Hardwicke et al., 2016). There is no consensus on what causes these poorer outcomes, and we have argued here for more research in to underlying developmental mechanisms. However, the patterns of early language development that we observed in this study may be an indication of poor speech development rather than language development per se. The questions for which all parents in both groups indicated fewer behaviors were related to speech development and the impact this can have on expressive language development. This included putting 2 different sounds together, using their voice to indicate a question, and the ability to imitate sounds, which was found to be the only question that significantly differed between the 2 groups (*P* = .02). Our study found that infants with a cleft of both the hard and soft palate were likely to exhibit fewer social communication and expressive language behaviors compared to those with a cleft of the soft palate only (*P* = .02); this was regardless of a diagnosis of RS. The impact of the severity of the cleft has been found to be related to speech outcomes in other studies. Persson et al. (2002) found that children with a cleft of the hard palate compared with those with a soft palate cleft were more likely to have retracted articulation (*P* < .05) at 5 years of age. Nyberg et al. (2010) found a significant difference between these 2 groups aged 4 to 6 years in terms of overall articulation skills (*P* = .04) and presence of glottal articulation (*P* = .02). Both studies included a few participants with RS in the hard palate cleft groups, but this was not the focus. In infants, the development of speech and early expressive language is interlinked, and the relationship between these 2 aspects of communication needs further research.

### Limitations

Limitations to this study relate to the sample. Small sample size is a common difficulty in studying a low incidence population and is a frequent criticism of studies which we were not able to address at the time the data were available, especially with regard to the RS group. This also meant that in an effort to include as much data as possible, we also had unequal group sizes with some older children in the iCP group. Analysis carried out with these outliers removed showed no differences in the results seen. This was a preliminary study and participant numbers in the Cleft Collective cohort continue to grow, allowing future larger investigations. There was also an element of selection bias. Although the return rate of questionnaires for the CC-SL study is excellent at 85%, those returned were disproportionately from mothers with a higher level of education. Mother’s level of education is known to be highly correlated with language outcomes and so is an important confounder in any study of child language development (Feldman et al., 2000; Reilly et al., 2007). Missing data were also an issue for the confounding variables. This was dealt with in the analysis in a variety of ways; no method changed the results.

## Conclusion

This study found no group differences between the early communication behaviors of infants with cleft palate with or without RS. It did find some divergence in more complex language skills. It also found some small differences in behaviors which may be related to speech sound development. It highlights a need for further research into this group. Larger matched group studies would enable investigation into the influence of other factors such as cleft width. Longitudinal studies would allow us to track the speech and language development of these children and see whether language problems persist. More prospective, multicenter studies would be highly beneficial to overcome the sampling difficulties when studying this low incidence-high need population group. Furthermore, research into the relationship between speech and expressive language development is also needed. This will inform clinical decision-making for intervention for all children born with cleft palate.

## Supplemental Material

Supplemental Material, sj-docx-1-cpc-10.1177_10556656211031877 - Early Communication Behaviors in Infants With Cleft Palate With and Without Robin Sequence: A Preliminary Study Click here for additional data file. Supplemental Material, sj-docx-1-cpc-10.1177_10556656211031877 for Early Communication Behaviors in Infants With Cleft Palate With and Without Robin Sequence: A Preliminary Study by Stephanie van Eeden, Yvonne Wren, Cristina McKean and Helen Stringer in The Cleft Palate-Craniofacial Journal
