# Supplementary material for: Early Communication Behaviors in Infants With Cleft Palate With and Without Robin Sequence: A Preliminary Study
Source: Cleft Palate Craniofac J. 2021 Jul 14;59(8):984–94. doi: 10.1177/10556656211031877 (PMC9272514; doi:10.1177/10556656211031877)
Supplement: Supplemental Material, sj-docx-1-cpc-10.1177_10556656211031877 - Early Communication Behaviors in Infants With Cleft Palate With and Without Robin Sequence: A Preliminary Study [file sj-docx-1-cpc-10.1177_10556656211031877.docx]

Appendix

LENA Developmental Snapshot (LDS) parent questionnaire and subdomains

| **Q #** | **Question** | **Subdomain** |
| --- | --- | --- |
| **1** | When parent talks to their child, child looks in the direction of the parent's voice | **Social communication** |
| **2** | Child vocalises or makes sounds in response to parent's smile or voice | **Social communication** |
| **3** | Child has different cries to indicate different needs | **Social communication** |
| **4** | Child expresses pleasure or displeasure by using sounds other than crying or laughing | **Social communication** |
| **5** | Child brings toys or objects to parent in their mouth | **Social communication** |
| **6** | Child laughs | **Social communication** |
| **7** | Child engages in 'vocal play' by producing a wide variety of sounds | **Social communication** |
| **8** | Child produces two or more vowel sounds, such as ah or ooh | **Social communication** |
| **9** | Child recognises their name (or nickname) | **Receptive** |
| **10** | Child shouts or makes sounds to get parent's attention | **Expressive** |
| **11** | Child imitates sounds parent or others make | **Social communication** |
| **12** | Child repeats two similar sounds together (not necessarily referring to a specific object or person) | **Social communication** |
| **13** | When parent says things to their child such as “want to get up?” or “bye‐bye” child responds by lifting his/her arms of waving | **Receptive** |
| **14** | Child puts different sounds together | **Social communication** |
| **15** | Child vocalises while gesturing to let parent know what they want | **Expressive** |
| **16** | Child says words besides “mama” or “dada” | **Expressive** |
| **17** | Child gives parent an object when asked | **Receptive** |
| **18** | Child follows simple 1‐step directions | **Receptive** |
| **19** | When parent names different objects, child points to them | **Receptive** |
| **20** | Parent can tell by the way the child's voice sounds that they are asking a question | **Social communication** |
| **21** | Child identifies basic body parts on himself/herself | **Receptive** |
| **22** | Child can say at least 10 meaningful words that parent consistently recognises | **Expressive** |
| **23** | Child points to objects named in books | **Receptive** |
| **24** | Child spontaneously repeats words that they have heard in conversation | **Expressive** |
| **25** | Child can follow 2‐step directions | **Receptive** |
| **26** | Child can understand the meaning of at least four action words without the use of gestures | **Receptive** |
| **27** | Child understands “what”, “where”, and “who” questions | **Receptive** |
| **28** | Child can name familiar objects in a room | **Expressive** |
| **29** | When parent points to pictures in a book, their child can name them | **Expressive** |
| **30** | Child understands 'location' words such as 'in', 'on', and 'out' | **Receptive** |
| **31** | Child can combine two or more words together to form simple phrases | **Expressive** |
| **32** | Child has at least a 50-word spoken vocabulary | **Expressive** |
| **33** | Child understands the concept of 'one' | **Receptive** |
| **34** | Child can follow 3‐step directions without getting distracted | **Receptive** |
| **35** | Child can say “I”, “me”, and “you” | **Expressive** |
| **36** | Child understands colour words | **Receptive** |
| **37** | Child has started to use size concepts | **Expressive** |
| **38** | Child uses sentences that are four words in length | **Expressive** |
| **39** | Child adds “‐s” to words to indicate “more than one” | **Expressive** |
| **40** | Child can tell you what to do with simple objects | **Expressive** |
| **41** | Child adds “‐ing” to the end of verbs to indicate ongoing action | **Expressive** |
| **42** | Child use the words “a”, “an”, and “the” | **Expressive** |
| **43** | Child can name common shapes such as circle, triangle, square and star | **Expressive** |
| **44** | Child understands concepts like “least”, “most” and “first” | **Receptive** |
| **45** | Child understands concepts like “tall”, “short” and “long” | **Receptive** |
| **46** | Child uses the plural pronouns “we”, “they”, “them” and “us” | **Expressive** |
| **47** | Child adds “‐ed” to the end of verbs to indicate an action that happened in the past | **Expressive** |
| **48** | Child can spontaneously produce sentences that are 10 or more words in length | **Expressive** |
| **49** | Child can name items that belong to a common category | **Expressive** |
| **50** | Child can retell a story or event with a beginning, middle, and end without using pictures | **Expressive** |
| **51** | When a parent names an object, child can describe two things about the object | **Expressive** |
| **52** | Child will ask parent about the meaning of words and then use the word in a sentence | **Expressive** |

Copyright © 2009, LENA Foundation. All Rights Reserved.
